# Comparison of Perioperative Outcomes for Complex Renal Tumors Between the Da Vinci and Hinotori Surgical Robot System During Robot-Assisted Partial Nephrectomy: A Propensity Score Matching Analysis

**DOI:** 10.3390/jcm14165850

**Published:** 2025-08-19

**Authors:** Daisuke Motoyama, Kyohei Watanabe, Yuto Matsushita, Hiromitsu Watanabe, Keita Tamura, Hideaki Miyake, Teruo Inamoto

**Affiliations:** 1 Department of Urology, Hamamatsu University School of Medicine, 1-20-1 Handayama, Chuo-Ku, Hamamatsu 431-3192, Japan; kyouhei2@hama-med.ac.jp (K.W.); yuto.m@hama-med.ac.jp (Y.M.); urohiro@hama-med.ac.jp (H.W.); ktamura@hama-med.ac.jp (K.T.); t-ina1@hama-med.ac.jp (T.I.); 2 Department of Developed Studies for Advanced Robotic Surgery, Hamamatsu University School of Medicine, Hamamatsu 431-3192, Japan; 3 Division of Urology, Kobe University Graduate School of Medicine, Kobe 650-0017, Japan; hmiyake@med.kobe-u.ac.jp

**Keywords:** complex renal tumors, Da Vinci, Hinotori, propensity score matching, robot-assisted partial nephrectomy, standardized mean difference

## Abstract

**Background/Objectives**: This study aimed to evaluate and compare the perioperative outcomes of robot-assisted partial nephrectomy (RAPN) for complex renal tumors performed using the novel Japanese Hinotori Surgical Robot System (HSRS) and the established Da Vinci Surgical System (DVSS). **Methods**: Of 484 consecutive patients who underwent RAPN at our institution, 126 with complex renal tumors were included in the DVSS group, and 48 such patients were included in the HSRS group. Complex tumors in this series were defined by the presence of at least one of the following factors: cT1b, completely endophytic, hilar, cystic, or ipsilateral multiple tumors. **Results**: Following 1:2 propensity score matching, 74 and 37 patients were included in the DVSS and HSRS groups, respectively. Post-matching, most covariates’ absolute standardized mean difference (SMD) was less than 0.1, indicating effective baseline imbalance correction. All RAPN procedures using HSRS were completed without conversion to open surgery, nephrectomy, or Clavien–Dindo ≥3 postoperative complications. No significant differences in major perioperative outcomes were observed between DVSS and HSRS, including operative time (178 vs. 186 min), console time (115 vs. 115 min; encompassing cockpit time for HSRS), warm ischemia time (15 vs. 15 min), and estimated blood loss (51 vs. 30 mL). Positive surgical margin rates (DVSS 1.4% vs. HSRS 5.4%) and Trifecta achievement rates (94.6% vs. 91.9%) were also comparable, with no significant differences. **Conclusions**: These findings suggest that, even in patients with complex renal tumors, RAPN performed using the HSRS can achieve perioperative outcomes comparable to those obtained with the established DVSS.

## 1. Introduction

In the past decade, robot-assisted partial nephrectomy (RAPN) has become the globally recognized standard surgical procedure for cT1 renal tumors [1]. While the Da Vinci Surgical System (DVSS) has long held a leading role in this field, the Hinotori™ Surgical Robot System (HSRS), developed in Japan in 2020, has recently been introduced into clinical practice as a next-generation surgical robot [2,3,4,5,6,7,8,9,10,11,12,13]. The Hinotori platform features a structural design akin to the Da Vinci Xi Surgical System (DVSS-Xi), with four multi-robot arms suspended from an overhead boom and an enclosed surgeon’s cockpit. This design allows surgeons to perform procedures in similar anatomical areas with a comparable operational feel to the DVSS, building upon established robotic surgical principles. Additionally, its multi-jointed robotic arms, equipped with eight joints or axes and a docking-free design, can reduce arm-to-arm interference and patient abdominal wall damage during surgery [10]. The system also features a comparatively compact design, optimized for the physical constraints of operating rooms in Japan.

A previous report from our institution demonstrated the feasibility and safety of RAPN using the HSRS, based on acceptable initial clinical results. Subsequently, we conducted a retrospective cohort study using propensity score matching to objectively assess its performance against the established DVSS [3]. This initial comparative study showed comparable perioperative outcomes between the two systems, suggesting the HSRS offers performance comparable to the existing platform concerning short-term surgical outcomes and safety during RAPN.

**Figure 1:**
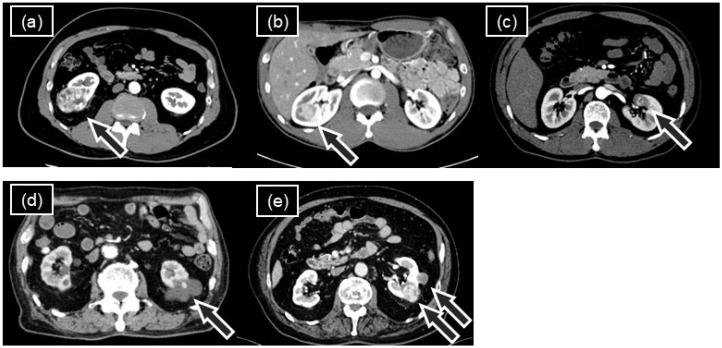
Representative axial views from preoperative contrast-enhanced abdominal computed tomography scans. In each image, arrows indicate renal tumors with complex features: (**a**) cT1b tumor, (**b**) completely endophytic tumor, (**c**) hilar tumor, (**d**) cystic tumor, (**e**) ipsilateral multiple tumors.

For complex renal tumors such as cT1b, completely endophytic, hilar, cystic, and ipsilateral multiple tumors (Figure 1), numerous previous reports have demonstrated the feasibility of RAPN using the DVSS with variable outcomes [14,15,16,17,18,19]. However, evidence supporting the HSRS’s use for such complex tumors remains limited. Given these considerations, this study focused on the perioperative outcomes of RAPN using the HSRS in patients with complex renal tumors, comparing them with DVSS outcomes using propensity score analysis to identify new supportive findings for the HSRS as an alternative to the existing system.

## 2. Materials and Methods

### 2.1. Patients

At our institution, RAPN using the DVSS-Xi was introduced in April 2016, and the HSRS was introduced in April 2022. From a cohort of 484 consecutive patients who underwent RAPN between April 2016 and March 2025, this study included 174 patients with cT1 renal masses exhibiting tumor-specific complexity. The study design was approved by our institution’s Research Ethics Committee (permission number: 21-091). Given its retrospective nature, individual informed consent was waived and patient consent was obtained via the opt-out method.

In this study, we defined complex factors based on preoperative contrast-enhanced CT and three-dimensional (3D) reconstructed images, evaluating the presence of the following morphological features: cT1b and completely endophytic, hilar, cystic, and ipsilateral multiple tumors. As previously described, renal tumors located in the renal hilum and physically contacting renal and/or segmental vessels were classified as hilar tumors [14]. To focus on real-world surgical difficulty, we included patients with tumors positive for at least one of these five factors, without relying on the RENAL nephrometry score [20].

### 2.2. Evaluation

Clinicopathological and perioperative data were retrospectively collected from the medical records of the 174 patients at our institution. Postoperative complications were graded according to the Clavien–Dindo classification system [21]. Routine contrast-enhanced CT was performed 3 to 5 days following RAPN to assess for urinary leakage and/or renal artery pseudoaneurysm [22]. This protocol was established after our institution experienced a severe case of a pseudoaneurysm that required emergency intervention. The routine screening aims to detect such silent but potentially life-threatening vascular complications before they rupture. In our country, this procedure is covered by health insurance for asymptomatic patients, supporting its use in our practice. In this study, Trifecta outcomes, widely recognized as a surrogate for successful partial nephrectomy, were defined by the concurrent achievement of three criteria, including a warm ischemia time (WIT) ≤ 25 min, negative surgical margins, and the absence of postoperative complications graded ≥ 3 according to the Clavien–Dindo system [23]. Furthermore, the margin, ischemia, and complications (MIC) score was calculated as a more stringent alternative to Trifecta, requiring an ischemia time < 20 min in addition to negative surgical margins and no Clavien–Dindo grade ≥ 3 complications [24].

### 2.3. Surgical Procedures

Detailed RAPN procedures at our institution using the DVSS and HSRS have been previously reported [3]. Standard surgical procedures at our institution included preoperative 3D reconstructed images, no routine ureteral stenting, a transperitoneal approach (except for dorsal hilar tumors or a history of intra-abdominal surgery), three robotic ports with two or three additional assistant ports, the same surgical technique for both robot models, total arterial clamping, cold knife incision around the tumor with a 5 mm margin under clear visualization, inner running suture, the early unclamping technique (standardized from the 53rd DVSS case in September 2017), soft coagulation, renorrhaphy, and Beriplast P Combi-Set (CSL Behring, Inc., Tokyo, Japan) as a hemostatic agent.

Particularly for cT1b tumors, due to their larger resected surface area, the hemostasis of small arterial vessels on the resected bed was performed with closely placed inner running sutures prior to unclamping. For completely endophytic tumors, a deeper vertical incision extending to the renal sinus was created during excision, and the resected specimen was then incised extracorporeally for visual tumor confirmation. For hilar tumors, the option of arteriovenous clamping was considered on a case-by-case basis, and any opened vessel walls during tumor excision were immediately repaired with continuous non-absorbable sutures. For cystic tumors, care was taken to prevent cyst rupture from excessive traction. For ipsilateral multiple tumors, the cutting technique was preferentially chosen over dissection around the tumors to facilitate the rapid excision of multiple tumors while considering the preservation of postoperative renal function. In cases of highly complex tumors with overlapping complexity factors, RAPN was conducted by experienced surgeons and assistants, prioritizing surgical safety.

In the pre-propensity score matching cohort, a total of eight surgeons performed the procedures using the DVSS, while six surgeons used the HSRS. Of these, only three surgeons performing DVSS procedures and two performing HSRS procedures had experience with more than 10 cases. The most experienced surgeon (H.M.) performed the largest number of procedures with both platforms (DVSS: 81 cases, 64.3%; HSRS: 19 cases, 39.6%), followed by surgeon D.M. (DVSS: 20 cases, 15.9%; HSRS: 12 cases, 25.0%). Four surgeons were only experienced with a single platform.

### 2.4. Statistical Analysis

All statistical analyses were performed using R ver. 3.1.1 (R Development Core Team, https://www.r-project.org (accessed on 18 July 2025)). A *p* value of <0.05 was considered statistically significant. Group differences were evaluated using Fisher’s exact test or the Mann–Whitney U test.

A 1:2 propensity score matching via logistic regression analysis with a caliper width of 0.20 was performed to adjust for baseline characteristics between the DVSS and HSRS groups. For this analysis, sex, cT1b tumor, completely endophytic tumor, hilar tumor, cystic tumor, ipsilateral multiple tumors, and the early unclamping technique were used as covariates. The quality of matching was assessed using the standardized mean difference (SMD). Post-matching, most baseline characteristics achieved an absolute SMD of less than 0.1, indicating good balance. However, the proportion of completely endophytic tumors showed an absolute SMD of 0.102, slightly exceeding 0.1. Nevertheless, this difference was considered clinically acceptable, and sufficient balance was confirmed for all other covariates. Therefore, the matched cohort in this study was considered adequately adjusted, and subsequent analyses proceeded.

## 3. Results

**Figure 2:**
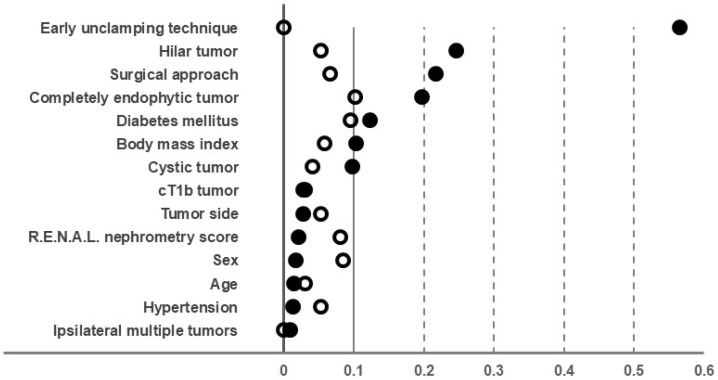
Changes in the absolute standardized mean difference (SMD) values of covariates before and after propensity score matching. Before matching (black circles), many covariates had an SMD greater than 0.1. Conversely, after propensity score matching (white circles), most covariates’ absolute SMD values improved to below 0.1. This indicates that the propensity score matching employed in this study effectively corrected baseline imbalances between the Da Vinci Surgical System and Hinotori Surgical Robot System groups, substantially enhancing their comparability.

This study included 174 patients with complex renal tumors who underwent RAPN at our institution. Of these, 126 patients were treated using the DVSS, and 48 received treatment with the HSRS. Table 1 summarizes the major baseline clinical characteristics and surgical procedures for these patients. In the entire cohort, several variables, including early unclamping, hilar tumor characteristics, and surgical approach, showed an absolute SMD exceeding 0.1. To address these baseline imbalances, a 1:2 propensity score matching approach was employed, adjusting for seven covariates, including the early unclamping technique (absolute SMD: 0.566), as described in the methodology. Following propensity score matching, the final analysis included 74 patients in the DVSS group and 37 in the HSRS group. Most baseline characteristics achieved an absolute SMD of less than 0.1, indicating adequate balance (Figure 2).

Table 2 compares the perioperative and pathological findings after propensity score matching between the DVSS and HSRS groups. All RAPN procedures in the HSRS group were completed without major peri- or postoperative complications (Clavien–Dindo ≥ 3), including renal artery pseudoaneurysm, urinary leakage, or conversion to nephrectomy or open surgery, despite tumor complexity. Pathological examination, however, revealed positive surgical margins in two patients. Overall, no statistically significant differences were observed in major perioperative outcomes, including operative time, console time (encompassing cockpit time for the HSRS), WIT, and estimated blood loss, between the DVSS and HSRS groups. Consequently, no significant differences were found in the achievement of Trifecta and MIC outcomes between the two groups. No significant differences in eGFR change were observed at either 1 or 28 days post-RAPN.

## 4. Discussion

The Hinotori platform is a novel surgical robot system developed in Japan, currently being introduced into clinical practice at numerous domestic institutions. As previously described, its characteristics include multi-jointed robotic arms with eight axes or joints featuring interference control, a compact setup with a docking-free design, and full high-definition imaging [3]. Furthermore, its most noteworthy feature is a configuration that builds upon the proven design principles of the DVSS-Xi, an operation unit with four robotic arms suspended from an overhead boom, an enclosed surgeon cockpit, and a vision unit. This design allows surgeons to perform a full range of urological procedures with an operational feel and approach comparable to that of the DVSS. This consequently enables leveraging extensive experience and know-how in robotic surgery, accumulated over more than a decade with the DVSS series while simultaneously benefiting from the various functional advantages of this new-generation system. In addition, the HSRS undergoes regular software and hardware updates. In 2023, operability was further enhanced by implementing a hand clutch and an assistant function for motion suspension [25].

However, comparative studies with the DVSS are currently limited to a few urological procedures, such as standard RAPN or robot-assisted radical prostatectomy [3,5,8,9,11]. To establish the HSRS as a viable alternative to existing systems in the future, accumulating evidence demonstrating its performance in complex and challenging surgeries is indispensable. From this perspective, the present study represents the first comparative report on whether the HSRS demonstrates sufficient capabilities for performing complex surgical procedures.

For analyzing the perioperative outcomes of complex RAPN procedures, an indicator accurately assessing tumor complexity is desirable. The RENAL nephrometry score is globally recognized as a simple, useful system for quantitatively assessing renal tumor complexity [20]. However, this score has inherent limitations leading to discrepancies in clinical practice, including its inability to numerically reflect the complexity of hilar or cystic tumors and its inadequacy for scoring ipsilateral multifocal tumors [14]. Considering these limitations and to accurately assess the true performance of both surgical robot systems on complex tumors, this study employed tumor complexity factors that better reflect actual surgical challenges, rather than the RENAL nephrometry score. Furthermore, we selected a propensity score-matched analysis to mitigate confounding biases from non-randomized system selection.

As a result, the Trifecta achievement rate was acceptable in both groups, exceeding 90% despite procedures targeting complex tumors. However, two cases of positive surgical margins in the HSRS group warrant caution. One involved a 58 mm clinical stage T1b renal tumor with preoperative imaging suggestive of non-clear-cell renal cell carcinoma (RCC). A partial rupture of its thin tumor capsule occurred during dissection, and final pathology revealed chromophobe RCC. In the other case, a patient presented with a 26 mm hilar renal tumor. Preoperative imaging again suggested non-clear-cell RCC, and a partial rupture of the thin tumor capsule occurred during tumor excision. Final pathology confirmed papillary RCC. While the numerical difference in positive surgical margin rates was not statistically significant, the detailed review of these two cases suggests the positive surgical margins were more likely to be a result of the inherent complexity of the tumors (e.g., thin tumor capsules, non-clear-cell histology) rather than a reflection of the HSRS platform’s precision or a surgeon’s adaptation issues. This highlights that meticulous dissection or excision, adapted to tumor-specific characteristics, remains paramount regardless of the surgical robotic platform. Consequently, this observation does not necessarily mean the HSRS platform is inferior to DVSS and supports our conclusion regarding the non-inferiority of HSRS.

To successfully manage complex tumors during RAPN, understanding the specific surgical techniques required for each complex factor is crucial. For cT1b tumors, their large diameter necessitates relatively wide dissection, which tends to prolong operative time [14]. Furthermore, as arterial bleeding from several points on the larger resection bed is often observed following early unclamping, definitive arterial hemostasis should be achieved by placing a thorough continuous suture within the renal parenchyma of the bed before releasing the clamp.

For completely endophytic tumors, which are often smaller than exophytic tumors despite having higher RENAL nephrometry scores, attention should be paid to their prolonged WIT due to excision difficulty [14,15]. To avoid incising the tumor, we routinely employ a deep vertical incision extending to the renal sinus for these tumors, regardless of size. Ideally, precise tumor location is identified by both preoperative and intraoperative ultrasonography; however, a certain number of tumors have indistinct margins. Therefore, extracorporeal incision of the specimen after excision to confirm tumor inclusion can be meaningful.

Hilar tumors necessitate preoperative discussion and strategic planning based on high-precision 3D reconstructed images. For example, if a hilar tumor abuts the renal vein or a major segmental vein, arteriovenous clamping is selected, requiring the additional clamping of any non-main renal arteries. Opened vascular walls during tumor excision are closed using non-absorbable sutures.

For cystic tumors, careless dissection or excessive traction can easily cause rupture, potentially leading to dissemination [17]. However, some prior research indicates that intraoperative cyst rupture does not worsen subsequent recurrence rates [26]. Nevertheless, for surgeons, including our team, achieving an en bloc excision without violating the tumor should remain a universal goal, even for non-cystic tumors.

For ipsilateral multiple tumors, the anticipated WIT is more than double the usual duration. Therefore, especially in patients with a solitary kidney, limiting excision to a maximum of two tumors might be the feasible extent to remain within WIT limits, considering potential renal functional damage [18,19]. Technically, WIT can be expected to shorten by consciously utilizing cutting motions more than dissection during multiple tumor resection.

These complex factors can overlap, dramatically increasing surgical difficulty. Therefore, for such highly complex cases, an experienced surgeon and assistant should be assigned to the procedure. This study demonstrated that the specific techniques for each of the aforementioned factors are sufficiently practicable with both the DVSS and the HSRS.

Beyond the scope of this study, the absence of a formal cost comparison and ergonomics evaluation is a notable point. Our study focused specifically on complex surgical cases; therefore, a comprehensive analysis of cost-effectiveness, including acquisition, maintenance, and instrument expenses, would require a broader, institution-wide dataset. Similarly, while a formal assessment of surgeon ergonomics and workload was not feasible, we recognize it as a critical factor for institutional decision-making. We acknowledge these important considerations and propose they are vital topics for future research as new robotic platforms become available.

This study has several limitations. First, its primary limitations include the inherent nature of a retrospective design, a relatively small cohort size (particularly in the HSRS group), an exclusive focus on short-term outcomes, variability introduced by multiple surgeons, and the lack of total cost comparisons. These findings necessitate validation in a future large-scale, prospective trial evaluating long-term oncological and functional outcomes, ideally encompassing data from a single surgeon and a comprehensive comparative cost analysis. Among these, the restricted sample size might not have yielded statistically significant differences for outcomes like estimated blood loss or positive surgical margins, introducing a potential Type II error where true differences could exist undetected. The varying levels of surgeon experience and proficiency with the two surgical platforms may have introduced a performance bias, as the procedures were performed by multiple surgeons, with only a few having extensive experience with both DVSS and HSRS. Therefore, the observed outcomes could be influenced by individual surgeon skill rather than the platform itself. Second, although the absolute SMD for the proportion of completely endophytic tumors in the propensity score matching was 0.102, marginally exceeding the 0.1 threshold, its impact on the primary analytical results is likely minimal, as adequate balance was achieved for most other covariates. Future research should explore this in more detail using more precise matching methods and sensitivity analyses. Finally, a potential learning curve bias must be acknowledged, as HSRS procedures were performed after extensive institutional experience with the DVSS. We believe, however, that this factor was unlikely to have significantly impacted outcomes. All early-phase DVSS cases, specifically the initial 52 cases before the early unclamping technique was introduced, were excluded from propensity score matching. Additionally, our previous reports demonstrated that proficiency in RAPN with DVSS can be achieved after a relatively short learning curve [27].

## 5. Conclusions

To our knowledge, this study represents the first comparative analysis of perioperative outcomes for complex renal tumors undergoing RAPN using the novel HSRS versus the established DVSS. In this propensity score-matched cohort, no significant differences were observed in key perioperative outcomes between these groups. Thus, our findings suggest that, even for patients with complex tumors, RAPN performed with the Hinotori platform yields perioperative outcomes non-inferior to those of the Da Vinci platform.

## Figures and Tables

**Table 1:** Patient characteristics before and after propensity score matching in complex renal tumor patients undergoing robot-assisted partial nephrectomy with Da Vinci Surgical System or Hinotori Surgical Robot System.

	Entire Cohort			Propensity Score-Matched Cohort		
	DVSS	HSRS		DVSS	HSRS	
	Group	Group	Absolute	Group	Group	Absolute
	(*n* = 126)	(*n* = 48)	SMD	(*n* = 74)	(*n* = 37)	SMD
Sex (%)			0.017			0.085
Male	83 (65.9)	32 (66.7)		49 (66.2)	23 (62.2)	
Female	43 (34.1)	16 (33.3)		25 (33.8)	14 (37.8)	
Age (%)			0.015			0.031
<75 years	99 (78.6)	38 (79.2)		55 (74.3)	28 (75.7)	
≥75 years	27 (21.4)	10 (20.8)		19 (25.7)	9 (24.3)	
Body mass index (%)			0.103			0.058
<22 kg/m^2^	31 (24.6)	14 (29.2)		22 (29.7)	12 (32.4)	
≥22 kg/m^2^	95 (75.4)	34 (70.8)		52 (70.3)	25 (67.6)	
Diabetes mellitus (%)	30 (23.8)	9 (18.8)	0.124	19 (25.7)	8 (21.6)	0.096
Hypertension (%)	56 (44.4)	21 (43.8)	0.014	36 (48.6)	17 (45.9)	0.054
Tumor side (%)			0.028			0.054
Right	70 (55.6)	26 (54/2)		38 (51.4)	20 (54.1)	
Left	56 (44.4)	22 (45.8)		36 (48.6)	17 (45.9)	
R.E.N.A.L. nephrometry score (%)			0.022			0.081
4–8	67 (53.2)	25 (52.1)		37 (50.0)	17 (45.9)	
9–12	59 (46.8)	23 (47.9)		37 (50.0)	20 (54.1)	
Tumor complexity						
cT1b tumor (%)	48 (38.1)	19 (39.6)	0.031	27 (36.5)	13 (35.1)	0.028
Completely endophytic tumor (%)	31 (24.6)	8 (16.7)	0.197	13 (17.6)	8 (21.6)	0.102
Hilar tumor (%)	57 (45.2)	16 (33.3)	0.246	34 (45.9)	16 (43.2)	0.054
Cystic tumor (%)	19 (15.1)	9 (18.8)	0.098	9 (12.2)	4 (10.8)	0.042
Ipsilateral multiple tumors (%)	5 (4.0)	2 (4.2)	0.010	2 (2.7)	1 (2.7)	<0.001
Surgical approach (%)			0.217			0.067
Transperitoneal	97 (77.0)	41 (85.4)		58 (78.4)	30 (81.1)	
Retroperitoneal	29 (23.0)	7 (14.6)		16 (21.6)	7 (8.9)	
Early unclamping technique (%)	106 (86.2)	48 (100.0)	0.566	74 (100.0)	37 (100.0)	<0.001

DVSS, Da Vinci Surgical System: HSRS, Hinotori Surgical Robot System: SMD, standardized mean difference.

**Table 2:** Comparison of perioperative outcomes in patients with complex renal tumors between the Da Vinci Surgical System and Hinotori Surgical Robot System groups after propensity score matching.

	Propensity Score-Matched Cohort		
	DVSS	HSRS	
	Group	Group	
	(*n* = 74)	(*n* = 37)	*p* Value
Operative time (min), median (range)	178 (100–446)	186 (119–297)	0.39
Console/cockpit time (min), median (range)	115 (48–237)	115 (73–228)	0.99
Warm ischemia time (min), median (range)	15 (8–37)	15 (6–35)	0.79
Estimated blood loss (mL), median (range)	51 (0–4195)	30 (3–415)	0.32
Resected tumor weight (g), median (range)	19 (3–170)	19 (2–90)	0.68
Histological subtype (%)			0.59
Clear cell renal cell carcinoma	53 (71.6)	26 (70.3)	
Other malignancy	11 (14.9)	8 (21.6)	
Benign tumor	10 (13.5)	3 (8.1)	
Positive cancer margins (%)	1 (1.4)	2 (5.4)	0.23
Intraoperative blood transfusion (%)	2 (2.7)	0 (0.0)	0.55
Conversion to open surgery (%)	1 (1.4)	0 (0.0)	1.00
Conversion to nephrectomy (%)	0 (0.0)	0 (0.0)	1.00
Renal artery pseudoaneurysm (%)	2 (2.7)	0 (0.0)	0.24
Urinary leakage (%)	0 (0.0)	0 (0.0)	1.00
Major postoperative complications (Clavien–Dindo 3 or 4)	2 (2.7)	0 (0.0)	0.56
Hospital stay after surgery (days), median (range)	7 (4–31)	7 (4–23)	0.57
Achievement of trifecta (%)	70 (94.6)	34 (91.9)	0.68
Achievement of MIC (%)	61 (82.4)	28 (75.7)	0.45
Change in eGFR (%), median (range)			
Postoperative 1 day	−15.2 (−68.6–15.8)	−20.3 (−54.0–4.0)	0.095
Postoperative 1 month	−7.8 (−65.9–15.8)	−11.4 (−32.5–12.8)	0.16

DVSS, Da Vinci Surgical System: HSRS, Hinotori Surgical Robot System: MIC, margin, ischemia and complications. eGFR, estimated glomerular filtration rate.

## Data Availability

The data presented in this study are available upon request from the corresponding author. The data are not publicly available due to privacy and ethical reasons.
